# The Impact of Patient Prejudice on Minoritized Female Physicians

**DOI:** 10.3389/fpubh.2022.902294

**Published:** 2022-07-05

**Authors:** Cheryl Dellasega, Jane-Frances Aruma, Natasha Sood, Doerthe A. Andreae

**Affiliations:** ^1^Department of Humanities, Penn State College of Medicine, Hershey, PA, United States; ^2^Penn State College of Medicine, Hershey, PA, United States; ^3^Division of Allergy and Immunology, Department of Dermatology, University of Utah, Salt Lake, UT, United States

**Keywords:** minoritized providers, female physicians, patient prejudice, provider mistreatment, bias

## Abstract

**Background:**

Patient bias and prejudice directed against physicians from diverse backgrounds is a frequent occurrence in healthcare. Female physicians have long experienced discrimination in the healthcare system based on their gender alone. The dynamic known as Patient Prejudice toward Providers (PPtP) is disproportionately affecting female physicians because it is frequently compounded by sexism.

**Aim:**

The goal of this study was to explore the impact of PPtP on female resident and attending physicians.

**Methods:**

Using transcribed one-on-one interviews from a larger study of PPtP affecting resident and attending physicians, ten interviews with female physicians (resident and attending) from diverse ethnic backgrounds and countries of training at a large academic medical center were analyzed. The authors independently reviewed the interviews using an iterative process within and across interviews to inductively identify repeating words, phrases, and concepts relevant to the study aim.

**Results:**

Demographics of the ten participants included age (mean 34.6 years), ethnicity (6 Asian, 2 Hispanic, 2 African), and country of training (10% IMG vs. 90% US trained). Four of the interviewees were residents and six were attendings. Themes that emerged from the analysis included experiencing “*A Gendered Continuum of Abuse,”* “*Establishing a Higher Standard of Competency,”* “*Overcoming the Stereotype of the White Male Physician,”* “*The Physicality of Self Identity,”* and “*The Need to be Protective of Minoritized Trainees.”* All participants agreed that these perceptions created an adverse environment at the workplace and impacted on patient care.

**Conclusions:**

Discrimination of physicians based on their gender or their race/ethnicity has been reported. This study highlights the compounded effects of patient prejudice on female minoritized physicians. Organizations and individuals should identify and implement strategies to address the impact of PPtP and sexism in order to create an environment where all women can thrive professionally.

## Introduction

Inequities have existed but gone unrecognized or unacknowledged within the American medical system for a very long time. In recent years, research has focused on suboptimal care provided to minoritized patients due to implicit bias within the health care system. Implicit bias, defined as unconscious associations and judgements that influence social behavior (1) may commonly include prejudice based on skin color, ethnicity, immigrant status, religion and gender (2).

Prejudice from providers toward patients is not the only form of bias that exists in the healthcare setting. A negative dynamic of prejudice and bias from patients and their families (PPtP, Patient Prejudice toward Providers) directed toward minoritized health care workers in the health care system in general has been documented in case reports and research studies (3, 4). PPtP arise when patients interact with providers whose perceived identity (e.g., gender identity, race, ethnicity) does not match their notion of an effective provider (5). PPtP can range from demeaning comments to outright refusal of care (6).

The increasing frequency of PPtP also at academic medical centers is important to document, especially where a diverse body of healthcare providers is providing care in less diverse settings. These occurrences pose a psychological toll on trainees, undermine learning opportunities, and may result in suboptimal patient care (5–7).

PPtP has a significant impact on the career and wellbeing of the affected physicians. Minoritized providers are more likely to experience these prejudices, and anecdotal reports highlight that these events are painful and emotional (6, 8–14). Importantly, these experiences compound over the course of a career, resulting in a high rate of burnout (6).

Retention rates of minoritized faculty in academic institutions are lower compared to white counterparts and Black assistant professors have the lowest promotion rates (15). Minoritized physicians receive the least NIH research funding (16, 17) and stark income differences between Black and white male physicians exist (18).

Sexism in medicine has been present since the first women pursued education as physicians (19, 20). According to the AAMC's *Physician Specialty Data Reports*, the percentage of women in the physician workforce has risen from 28.3% in 2007 to 36.3% in 2020. Despite this steady rise, bias based on gender, as well as sexism against women in medicine has continued to be rampant (21).

While women currently constitute over half of medical school students (21, 22), they continue to occupy fewer leadership positions after graduation (23–25) and are more likely to leave the profession than their male counterparts (26–28). Female physicians continue to be underrepresented in specialties that offer higher incomes and prestige (OECD, 2021). Implicit bias within the healthcare system against female physicians in general and minoritized female physicians in specific is affecting wellbeing and career advancement on many levels (29). This is being reflected in measurable outcomes like lower incomes of female vs. male physicians for the same work (18, 30) but also less tangible measures like perceived lack of support and respect from faculty reported by minoritized female students (31).

Biases against women in medicine, especially minoritized female physicians, may also be perpetrated by patients. As the physician workforce is becoming increasingly more diverse, patients are more likely to encounter physicians that may not conform to certain pre-conceived notions of what a physician should look like, leading some patients to react with biased behavior in response to such encounters. Despite this being the lived experience of many minoritized female physicians, there is a paucity of scholarship on these events. A few anecdotal reports suggest that women of color or from different countries of origin are at special risk to prejudiced behaviors from patients and male minoritized physicians have reflected on the additional hurdles their female minoritized colleagues face (unpublished results, DAA and CD). In addition to the discrimination female physicians experienced from their patients and their families many report the important role of colleagues, supervisors and the institution in responding to these incidents.

The lack of data on the dimensions of these encounters makes it challenging to create policies or training programs to address.

Institutional policies to support trainees, faculty, and staff in managing PPtP is critical (32). More specifically, academic medical centers need data on the nuances of gendered PPtP, how trainees respond to these behaviors, and what institutional factors influence their response to these incidents.

It was the purpose of this research to explore the gendered impact of PPtP on female resident and attending physicians. A qualitative design was chosen because it captures details of lived experiences that surveys do not; that is, it “considers why individuals think or behave the way that they do and how they come to understand these complex thoughts and actions within their lives.”

To obtain rich data on reactions, attitudes and beliefs, a phenomenological framework was developed to capture the lived experiencing of a group of individuals (participants) around a specific phenomenon, (in this case, PPtP) (33, 34).

## Methods

To insure rigor and trustworthiness, the Consolidated Criteria for Reporting Qualitative Research (COREQ) was used (35).

### The Research Team

The investigators consisted of one physician researcher who also is an International Medical Graduate and Immigrant to the US (DAA), a faculty researcher with expertise in qualitative methods (CD) and four first- and second-year medical students who received 4 h of training on interviewing skills. All members of the team were female and three came from minoritized backgrounds.

### Participants

After receiving IRB approval, fliers were circulated to clinical and educational departments within an academic medical center. Additionally, word-of-mouth and snowballing were used to recruit the final sample of 11 resident physicians and 15 attending physicians .6 of the residents and 1 of the attending physicians 2 who volunteered did not schedule interviews due to a lack of time. Interviews with the 10 female physicians of the larger study (4 resident and 6 attendings) were analyzed separately for this study. Data on the resident sample has been published elsewhere (3). The participants self-identified as minoritized by virtue of their ethnicity, country of origin, or religious background. All physicians who volunteered to participate were currently employed within the same health system, although some of their reported experiences with PPtP occurred at other institutions. Prior to beginning each interview, basic demographic data was collected from each participant. For a description of the sample, see Table 1.

**Table 1:** Demographics.

**Item**	***N***	**%/Mean**	**Range**
Age	10	34.6	24–55
**Credential**			
Resident	4	40	
Attending	6	60	
**Practice population**			
Adult	3	30	
Pediatrics	1	10	
Both	6	60	
**Specialty**			
Medical	9	90	
Surgery	1	10	
**Country of birth**			
US	3	30	
Not-US	7	70	
**Citizenship**			
US	8	80	
Other	2	20	
**Ethnicity**			
Black/AA	2	20	
Asian American	6	60	
Latinx	2	20	
**Medical school**			
US	9	90	
IMG	1	10	
Time at current institution	10	4.86	1.5 mos-6 Years

### Data Collection

Interviews were scheduled at a time and place convenient to participants. Prior to Covid, the research assistants met with interviewees in a quiet private space where they would not be interrupted or overheard. During the Covid pandemic, zoom interviews were used, again at a time of the participant's choosing and in a space that ensured confidentiality and privacy.

Each participant was interviewed once, with interviews lasting, on average, 60 min. All interviews were taped, stored in a secure location and transcribed from the audio-file including pauses and emphases.

An 11-question interview guide (see Appendix A) was developed for use in a preliminary study of PPtP among resident physicians. The questions were developed based on a review of the scholarly literature, input from experts, and discussions with individuals who identified as minoritized providers.

Data saturation, which was defined as the point when additional interviews collect no new themes or information needed to address the research question, was reached after 11 interviews for resident data and 15 interviews for physician data (36). Our sample size is within range compared to the existing literature reporting that within the first six interviews most themes are reported (37). Our resident sample size was smaller as the residents represented a more homogeneous group, similar in age and training stages. No repeat interviews were offered.

### Data Analysis

The co-investigators (DAA, CD) reviewed each transcript separately, reviewing within and across interviews for repeating words, concepts and phrases that captured the lived experience of females who had been in situations of PPtP. Once the independent analysis had been performed, the investigators met to further analyze data relevant to participant experiences.

Data were merged into categories and then five themes that emerged from the data. The Kappa coefficient of agreement between the investigators was 0.88. After the five themes were identified, no new themes occurred and the team returned to the data in order to identify exemplar statements that best illustrated (and confirmed) the themes. Finally, validation of themes through independent reviewers (NS, JA) was conducted. Participants did not review and comment on extracted themes.

## Results

### Demographics

The 10 female interviewees ranged in age from 24 to 55 years with a mean age of 34.6 years .7 (70%) of the interviewees were born outside the United States, but 8 (80%) were US citizens. The self-reported race/ethnicity was 2 (20%) African-American, 6 (60%) Asian-American and 2 (20%) Latinx. All, but one participant had attended medical school in the United States .4 interviewees were in residency training and 6 participants were attending physicians. The majority of participants, 6 (60%), treated both pediatric and adult patients in their daily practice. One participant treated exclusively pediatric patients and 3 (30%) treated adult patients only. One interviewee reported working in an exclusively surgical specialty .9 (90%) of the interviewees were training or working in a medical specialty.

For details please refer to Table 1.

**Five themes emerged from the data and are described in** **Table 2**.

**Table 2:** Thematic analysis.

**Theme**	**Exemplar**
A Gendered Continuum of Abuse	“And the resident made a comment like, “Oh, I'm so excited we have this great team”, and he was referring to the male medical student and his male senior and had, like, his arms around both of them. Meanwhile I was walking in front of them and turned around and said, “What am I, chopped liver?” Because he hadn't said my name but he had included the other two's names in the team, but not mine.” “But so many times in residency patients would say to me, “Can I have a pillow?” “Can you help clean up this mess?” And I would be with a group of other residents but I would be singled out. And I don't know if that's because of my ethnicity, or if it was because of my gender, or if it was both so I think they just assumed that I was either a nurse or I was housekeeping. And they wouldn't ask my male colleagues.” “And then also small things to be confused for the nurse because you're female. Like it doesn't matter how many times you introduce yourself as a doctor, you come back the very next day and you're the nurse. I'm like, how can I change my status from day to day? It's like I'm the doctor and I told you that.” “So, from nursing I think that's the biggest most prohibitive and toxic way. First of all, in the medical ICU: if male residents are kind to nurses in our medical ICU they're lauded and loved. Versus, I know when I'm smiling and nice to them, they actually don't trust me and they roll their eyes at me.” “And there were countless times when I would walk into the room and they would get off the phone by saying, ‘Oh, I have to go, the nurse is here.” Or they would ask me, “Oh, are you a nursing student?” I would say, “No, I'm a medical student.” And they'd say, “Oh, what kind of nurse are you going to be?”, after I just told them I was a medical student.”
Establishing a higher standard of competency	“So, I think they feel, not all, but there are a few people who are just, they try to take advantage of you, thinking that you might not be strong enough or confident enough with what you're doing.” “…in the past, there were a few comments where in, um, some of the patients were pleasantly surprised that my spoken English and accent was not as bad as they perceived…so I've seen or sensed that some-some patients when I go to see them into the room they probably didn't expect me or they seem surprised, um, and they're not as forthcoming initially but once I start talking and then kind of converse with them and give information they seem to be more open…I've seen that happen time and time again.” “…I've had experiences of being treated both by attendings and by patients, ah, as being, like, less smart or less confident or less capable because I think being a woman and being a colored woman…” “…And so, I think the battle with being a black physician is the hurdle of credibility…it can make you develop a complex because you already have imposter syndrome, you're probably the only black person in the department or in the residency…. we have such an additional psychological burden that [the] majority of people will never understand…” “Having my decisions rudely questioned or responded to by nurses is a big one for me. In general, the way nursing is and the way they ask for things, the way they page for things, and the way they differentially treat male residents versus female residents. […] And I've asked my male colleagues if this is what they run into and they say, “No, they just get it for me or do it for me”.”
Overcoming the stereotype of the white male physician	“…, it's not every day, so I didn't, um, at least two or three times a week…I think it could be worse…if my gender was different and nothing else about me changed, just my gender, would it be worse?” “…it seems that it's easier for patients to feel…ah… comfortable speaking to someone that looks like the stereotypical physician, which at the current moment our stereotypical physician is a white male…” “…You know, it's when somebody judges you based on something you can't help, like your skin tone or your gender or your sexuality, it can really throw you and now you're not there 100% for the rest of your patients.” “And then I had an instance of somebody that just clearly seemed to be fixated on my intern, who was, like, an all-over white male on being the doctor when I was the senior resident and trying to get his information, so I guess, just gravitating toward the white doctor in the room …” “So, he's like, “Hi I'm < first name>, < first name, last name>.” He then introduces everybody by first name. So, one day I actually came out of the room and I was pissed off and I was like, “Hey, why are we introducing ourself by first name?” And he's like, “It sets rapport with the patient.” I'm like, “Yeah, that's easy for you to say. You're a tall white dude […] you walk into the room and everybody knows you're a doctor. I walk in and people think I'm a nurse”.”
Physicality of self identity	“I think they're very pleasant but as they come and approach face-to-face then they're like, “Oh, [she] has eye brows which are not very well done” […] and then, “Oh, this girl looks a little different”. And then I see their reactions in the eyes, [they] look a little disgusted… So, I do feel that the young, probably very young, white nurses they do feel that.” “…being confused from a nurse I always took it with pride to be honest… being asked to do things as a short Latina is very common place and being asked to do things that you know white male counterparts would never be asked to do from patients…” “For example, like one or two say, “You always change your hair”. I say, “Yeah, because I have the ability to change my hair, to wear braids or whatever. Like, why are you saying this in front of the patient? Like why?” “He physically looked over me. Like I was standing closer to him. I'm 5'2” and he's probably 6'2”, like, tall enough that there's a substantial height different between the two of us. But I wasn't like within a foot that he had to look straight down at me to see me. There was enough room that I was within his field of sight, but the two 3^rd^ years were standing behind me and he very visibly looked over and past me toward them. He walked over to them and clapped one of them on the back like, “Oh are you gonna do this?”
	“A lot of patients ask if I'm pregnant because I have a prominent belly. We have overweight white female attendings and I've never seen them ask if they're pregnant. So, I don't know if my minority played into patients asking if I'm pregnant, but it happens a lot.”
The need to be protective of minoritized trainees	“…I was the attending and he [Asian resident] came out and presented to me and then I went in and the patient was like, “I did not understand a word that [expletive] doctor said to me.” And this guy [resident] was born and raised in New York.so I said, “Actually, he speaks English very well, so I'm surprised you did not understand what he was saying”.” “I think, if it were to happen to one of my trainees, either medical students, residents, or fellows, I think I feel a lot more comfortable and I care a lot less about offending people. I think I would just outright stop it and explain to them how it's inappropriate. And so, I think if it it's coming from a patient, I think I would be a lot more bold than I used to be. I was a lot more timid about things, and again it probably comes with age and being more jaded…” “When I'm with students, it's a little different…I know the students and residents have these racial interactions and encounters…but the comments I see toward, you know, students and residents, particularly residents, I wouldn't say students, probably more residents, um that I would guess, are more along that sexual, you know, that gender bias, you know so ageism, right? You know you're too young to know what you're talking about.” “… I mean I can't step in for my intern because I'm like the same background as her and it's kind of weird so I page her out of the room.” [When PPtP occurs]

Themes that emerged from the analysis included experiencing the following:

### A Gendered Continuum of Abuse

Many of the behaviors interviewees described were covert, such as ignoring, undermining, negating or excluding the Female Medical Doctor of Color (FMDOC). Other more overt behaviors included refusing care from the FMDOC or commenting on appearance. Often, these PPtP behaviors occurred when there were men in the room of lesser status than the female resident or physician. Another overt behavior which was pervasive was being mistaken for a nurse (mentioned 9 times) or ancillary or other personnel. It was observed that minoritized male physicians were not treated in a similar fashion.

These behaviors generated anxiety for women, who anticipated mistreatment. In addition to being targeted by sexist comments, they were also aware of their minority status as a possible source of discrimination. In comparing the female resident with the female attending physician perspective, the perception of abuse did not stop after formal training ended but may have a diminished impact over time.

### Establishing a Higher Standard of Competency

The double threat of being female and from a minority background continued to place interviewees in a position of dealing with prejudice and bias from patients and sometimes coworkers who felt they were inferior because of both their gender and background. This manifested as challenging decisions, assuming incompetence, and increased pressure to justify medical decisions and actions, Comments suggested that participants sensed they were being held to a higher standard than their male counterparts.

### Overcoming the Stereotype of the White Male Physician

One pervasive theme was the assumption by both patients and providers that the only individuals who could be doctors were white males. In some ways, this may relate to the many complaints that female residents and attendings were often mistaken for and treated like a nurse.

With white males as the default representation of a physician, interviewees were confronted by instant facial expressions of suspicion or mistrust when they entered a patient's room. Interviewees noted how their male colleagues often failed to address sexism, even encouraging patients to call the team members by their first names which made clear identification of the individual's credentials less clear and often led to the female provider being mistaken for a nurse.

Even age presented a problem, with some interviewees reporting they were told they looked “too young” to be a physician, in addition to overt suggestions that they were also the wrong gender and skin color. When a patient rejected them overtly or covertly, participants wondered which combination of age, race, and gender led to the behavior.

### Physicality of Self Identity

In comparison with male residents and physicians who were interviewed previously (3) (and unpublished results), FMDOC were acutely aware of the way their physical experience impacted on prejudice. Skin tone, height and weight, body shape, and hair styling were all mentioned as ways patients negated their professional identity, factors that did not apply to male physicians.

The report of these exchanges rendered the women we interviewed feeling inadequate and “less than,” unlike the males we interviewed in the preliminary study (3).

### The Confidence to Be Protective of Minoritized Trainees

FMDOC expressed an obligation or inclination to intervene on behalf of trainees similar to themselves, whom they considered as especially vulnerable to PPtP because of their status as a trainee. This support of junior minoritized physicians was reported by both attendings and senior residents. Many reported feeling an obligation to intervene based on their own experiences with PPtP where they were unsupported. Due to their relatively more senior position and role, minoritized female attending physicians described feeling more confident to intervene on behalf of more junior physicians. Many still felt more difficulty responding on their own behalf.

## Discussion

All FMDOC in our study reported having experienced multiple situations of prejudice and discrimination from patients because of their race/ethnicity and gender. In addition to experiencing bias from patients and their families, prejudice and discrimination from colleagues and nurses has also been reported. This dynamic contributed to an adverse workplace environment and had a potential adverse effect on patient care. Thus, participants described a complex and negative situation as a consequence of their gender and race/ethnicity.

While gender inequities have existed since early societies and have persisted even though women have been more equally represented in the modern workforce (29), many female physicians report they are still treated with bias and prejudice because of their gender. This experience was multiplied and compounded for minoritized female physicians.

In our study the interviewees frequently reflected when describing negative patient encounters and wondered to what extent the discrimination was due to their gender alone or also based on their race and ethnicity.

These “gendered expectations” contribute to the significantly greater rates of burnout among female physicians (38). Our interviewees' reports of having to fulfill different expectations are reflected in the described model of patient-provider relationships that women are expected to offer more empathy and time in therapeutic relationships, leading to longer interactions and increased demands on time management. Especially the attending physicians we interviewed reflected on the significant impact this dynamic has on their careers and wellbeing. As Butkus et al. state: “Women in medicine face other challenges, including a lack of mentors, discrimination, gender bias, cultural environment of the workplace, imposter syndrome, and the need for better work–life integration” (39).

This not only impacts the provider patient relationship but many interactions between colleagues. For example as one of our interviewed residents (resident 5) described: “He physically looked over me. Like I was standing closer to him. I'm 5'2” and he's probably 6'2, “like, tall enough that there's a substantial height different between the two of us. But I wasn't like within a foot that he had to look straight down at me to see me.” Many female physicians are familiar with day to day experiences including men “talking over” female colleagues in meetings, women being addressed by their first names while men are addressed as “Doctor,” as well as women facing obstacles in promotion and being excluded from positions of leadership (40).

Some other common manifestations of sexism and inequity as experienced by women in medicine include subjection to sexist jokes in class; sexual harassment by clinicians, faculty, or patients; weaker recommendation letters than men for positions on medical school faculty; lower income than men; less recognition; tendency toward choosing lower paid medical fields; stigma associated with pregnancy especially during training and inadequacy of maternity leave options (20, 41, 42).

The experiences described by female physicians of all races and ethnicities resemble experiences described by minoritized physicians of both genders in our initial study interviews (3). However, it is noteworthy that in our original study, minoritized male physicians commented on the greater likelihood that their female counterparts would be targeted by patients more strongly and more frequently.

In our interviews many of the physicians reflected on being vulnerable to negative patient ratings and having to “overcompensate”. Kauff et al. examined ratings of 140,000 physicians on a German website for health professionals and discovered that females and physicians with a migration background (identified by foreign names) were consistently rated lower than males. They suggest that although medicine is a prestigious profession, those within who are from low status groups (women and immigrants) may be easily targeted and perceived in a negative way (43).

While our study specifically focused on prejudice and bias from patients many of the interviewees also reported being subjected to bias from other members of the health care team. Many of the situations described in our interviews were microaggressions such as looks because of a difference in dress or appearance to ignoring the physician and addressing the male physicians in the room. A meta-analysis by Filut et al. looked at workplace discrimination against physicians of color and found that prejudice was higher against Black and female physicians of color (44). Women of color faculty face significant obstacles such as promotion bias, implicit bias, financial constraint as well as unequal distribution of research non-clinical time. The promotion rate for African Americans (18.8%) and Latinos (23.5%) is less than that of whites (30.2%) when advancing from associate to full professor (45).

Schmitt et al. conducted two meta-analyses which explored the impact of perceived discrimination and found they supported a negative impact on psychological wellbeing (46). This can lead to burnout and turnover (20). For the FMDOC in our study, the experience of prejudice from the very patients they were assigned to care for had both an ethical and health impact. First, their personal wellbeing was diminished, but also, questions about continuing to provide care for a patient who exhibited negative behaviors toward you was troublesome. Interviewees reported reflecting on the ethical implications of altering care because of the patient's hostile behavior as opposed to suppressing their own wellbeing and continuing the therapeutic relationship. Some interviewees formulated their own strategies, such requesting to be paged out of the room, doing the minimal face-to-face work possible to still provide competent care, and when confronting patients about their comments, doing so with humor.

In addition to suggesting further research, Rodriguez et al. offered these strategies to support women such as the ones in our study: (1) Facilitate leadership positions to minoritized female physicians through “family-friendly” policies and mentorship programs, (2) Create cohorts through targeted funding and programs that build community through affinity groups, (3) Promote accountability through collection of data that tracks sensitive outcomes such as promotion and tenure (47).

The range of prejudice and bias expressed by patients ranged from microaggressions such as seemingly curious questions or comments to overt verbal or physical rejection, such as refusing to see a certain physician and walking out of the room.

A qualitative study conducted by Wheeler et al. (6) described some experiences of physicians with biased patient behavior. Spanning from explicit and degrading remarks to complete refusal of care, these behaviors were reported to exert challenging emotional and moral distress on both the targeted physicians and bystanders (6). Minority physicians are significantly more likely to experience this (10). A qualitative study of minority resident physicians by Osseo-Asare et al. reported that these physicians were frequently mistaken for non-medical hospital staff by patients and patients' families (48). Nearly all of our FMDOC interviewees reported incidents of being mistaken for nurses or other support staff in the hospital. Many described strategies to be perceived as the physician like avoiding certain clothes that might have the same color as the cleaning personnel or always introducing themselves with their full name and title.

These behaviors took on a unique impact for interviewees because they often involved physical appearance, just as females viewed themselves as unique in terms of their size, skin color, or country of origin.

The consequences of these prejudiced behaviors had an even greater effect during the residency years for FMDOC, who were also vulnerable due to their status as a trainee. Several of the female attending interviewees reflected back on their training years and suggested they had learned to cope with PPtP or been focused on building their practice and just ignored it and the psychological harm it causes.

Options for reacting were limited at the resident/trainee level. A cross sectional national survey of general surgery residents showed that residents who reported experience of and exposure to discrimination, abuse or harassment had increased likelihood to suffer symptoms of burnout and suicidal thoughts, when compared to their counterparts with no reported exposure to mistreatment (49). Female attendings and senior residents felt very strongly that they should use their relatively higher status and experience to protect younger trainees and lessen the negative impact of PPtP on the next generation of female minoritized physicians.

### Limitations

The study analyzed interviews with 10 female physicians. All participants reported a female gender identity but no additional demographic data was extracted on biologic sex vs. gender identity or LGBTQ + status. These factors could add an additional area of bias, which has been reported in the literature (50), and is an important consideration for future studies.

Additionally, although our sample size is qualitative and limited to one institution (although participants had rotated through many others), they are not generalizable. Given the homogeneity of the sample (all female, all at the same institution, and all similar in demographics), it is not surprising that themes began to emerge in the first 6 interviews, with additional interviews providing no new perspectives (37).

Finally, qualitative methods come with limitations. We choose a phenomenological approach to capture the lived experience of these FMOC because there was no existing statistical measure and our intent to gather rich, in-depth data. Since the qualitative component has been completed, we have developed a survey on PPtP which is being tested nationally for reliability.

## Conclusion

In our preliminary study of both male and female physicians and residents, one male attending interviewee remarked, “I imagine all this is even worse for women.” The results of our analysis confirmed this. Most prominent was the struggle to overcome the stereotype, held by both patients and colleagues, that the white male doctor was still the optimal and preferred provider. Denying the female's authority or legitimacy, asking for a “different” doctor, and statements about wanting an “American” doctor impacted the majority of participants.

Staying true to one's physical appearance, culture and gender while overcoming negative stereotypes and being recognized as the physician and partner in care to build the trusting relationship with the patient remains a daily struggle. This cannot be solved by the affected physicians alone but requires strategies on the organizational level.

## Data Availability Statement

The raw data supporting the conclusions of this article will be made available by the authors, without undue reservation.

## Ethics Statement

This study was reviewed by the Penn State Health Review Board and considered exempt STUDY00016826. Written informed consent for participation was not required for this study in accordance with the national legislation and the institutional requirements.

## Author Contributions

CD and DA: study conception and design. CD, DA, J-FA, and NS: data collection and draft manuscript preparation. CD, DA, and NS: analysis and interpretation of results. All authors reviewed the results and approved the final version of the manuscript.

## Funding

This project was funded through the Junior Faculty Development Program at Penn State 2018-2021, awarded to DA.

## Conflict of Interest

The authors declare that the research was conducted in the absence of any commercial or financial relationships that could be construed as a potential conflict of interest.

## Publisher's Note

All claims expressed in this article are solely those of the authors and do not necessarily represent those of their affiliated organizations, or those of the publisher, the editors and the reviewers. Any product that may be evaluated in this article, or claim that may be made by its manufacturer, is not guaranteed or endorsed by the publisher.
